# Effectiveness of Drug Treatments for Lowering Uric Acid on Renal Function in Patients With Chronic Kidney Disease and Hyperuricemia: A Network Meta-Analysis of Randomized Controlled Trials

**DOI:** 10.3389/fphar.2021.690557

**Published:** 2021-08-03

**Authors:** Xiang Liu, Yuxuan Qiu, Duohui Li, Jiaxing Tan, Xiuping Liang, Wei Qin

**Affiliations:** ^1^ Division of Nephrology, Department of Medicine, West China Hospital, Sichuan University, Chengdu, China; ^2^ West China School of Medicine, Sichuan University, Chengdu, China; ^3^ Division of Ultrasound, West China Hospital, Sichuan University, Chengdu, China

**Keywords:** hyperuricemia, chronic kidney disease, febuxostat, allopurinol, benzbromarone

## Abstract

**Background:** Hyperuricemia is very common in patients with chronic kidney disease (CKD); the role of hyperuricemia in the occurrence and progression of kidney disease remains an interesting and unresolved issue for nephrologists, and whether urate-lowering therapy (ULT) is warranted in CKD patients is still in controversy. To summarize and compare the clinical outcomes and adverse events (AEs) of three common ULT drugs, we performed a systematic review and network meta-analysis of randomized clinical trials (RCTs).

**Method:** PubMed, MEDLINE, Clinical Trials.gov, EMBASE, and the Cochrane Central Register of Controlled Trials electronic databases were searched. The network meta-analysis was performed using the “gemtc 0.8-7” and its dependent packages in R software. The primary outcome was the change of renal function and uric acid; creatinine, proteinuria, blood pressure, and adverse events were assessed as the secondary outcomes.

**Results:** 16 RCTs involving 1,943 patients were included in the final network analysis. Febuxostat, allopurinol, and benzbromarone were not found to exert superior effects over placebo upon renoprotective effect. With respect to lowering urate, the three drugs showed to be statistically superior to placebo, while febuxostat could better lower urate than allopurinol (MD: −1.547; 95% CrI: −2.473 to −0.626). It is also indicated that febuxostat was superior to placebo at controlling blood pressure, while no differences were observed when allopurinol and benzbromarone were compared to placebo. These results are stable in subgroup analysis.

**Conclusion:** There is insufficient evidence to support the renoprotective effects of the three urate-lowering agents in CKD patients with hyperuricemia; febuxostat shows a tendency to be superior to allopurinol on lowering the decline of eGFR and increment of proteinturia, but the difference does not reach a statistical significance. Regarding its urate-lowering effect, febuxostat appears to be a satisfactory alternative to allopurinol and benzbromarone, and can control blood pressure better.

## Introduction

Chronic kidney disease (CKD) is a public health problem that causes substantial morbidity and mortality because of its potential to progress to end-stage renal disease and promote high risk of cardiovascular events (Collaboration, 2020). Progression of CKD can result in decreased quality of life, increased medical expenses, and end-stage renal failure. Therefore, it is imperative to identify therapies that can slow the deterioration of kidney function.

Hyperuricemia is very common in CKD patients because of reduced urinary excretion of uric acid (Reginato et al., 2012). Hyperuricemia was also reported to promote development and progression of CKD (Jalal et al., 2013; Uchida et al., 2015). However, whether hyperuricemia is an indirect marker of impaired kidney function or plays a causative role in progression of kidney disease, or both, remains an interesting and unresolved issue for nephrologists (Sampson et al., 2017). The role of uric acid in the development of CKD and whether urate-lowering therapy (ULT) is warranted for its treatment are controversial issues (Tiku et al., 2018; Sato et al., 2019; Steiger et al., 2020).

Xanthine oxidase inhibitors are considered the primary class of ULT for patients with CKD. Although there are concerns over use of allopurinol (it induces fatal hypersensitive reactions and nephrotoxicity), it is still recommended as first-line therapy (Sato et al., 2019). However, these concerns may lead to allopurinol underdosing, resulting in poor control of hyperuricemia, and fewer studies were conducted on the use of allopurinol at sufficient doses (≥300 mg per day) (Stamp et al., 2011; Sato et al., 2019). An alternative, novel, and potent nonpurine selective xanthine oxidase inhibitor is febuxostat. It is well tolerated (Tiku et al., 2018) and does not require dose modification. Furthermore, it has been increasingly studied and was shown to exert satisfactory urate-lowering and renoprotective effects (Shibagaki et al., 2014). Another drug used for ULT, benzbromarone, is extensively prescribed in South America and Asia (Lee et al., 2008). Determining whether these common drugs used as ULT exert renoprotective effects when compared with placebo or usual therapy has been the subject of prior meta-analyses (Bose et al., 2014; Kanji et al., 2015; Liu et al., 2018; Zeng et al., 2018). However, direct comparisons of allopurinol, febuxostat, and benzbromarone remain scarce.

To summarize and compare the clinical outcomes and adverse events (AEs) associated with these three common drugs, we performed a systematic review and network meta-analysis of relevant randomized clinical trials (RCTs).

## Materials and Methods

### Data Sources and Search Strategy

This systematic review was performed according to the Preferred Reporting Items for Systematic Reviews and Meta-analyses (PRISMA) statement extension for network meta-analysis (Hutton et al., 2015) and PRISMA guidelines (Moher et al., 2009). PubMed, MEDLINE, Clinical Trials.gov, EMBASE, and Cochrane Central Register of Controlled Trials electronic databases were comprehensively searched up to June 1, 2020. Search terms are shown in Supplementary Table 1.

### Selection Criteria

All non-RCTs were excluded and language was restricted to English. Selected RCTs were those that 1) included CKD patients aged 18 years or older with hyperuricemia and who were being treated with the specified interventions (allopurinol, febuxostat, benzbromarone, placebo, or usual therapy); and 2) reported changes in renal function through measurement of the estimated glomerular filtration rate (eGFR), creatinine, or proteinuria. Considering that the definition of hyperuricemia has not reached a consensus at the current stage (Bardin and Richette, 2014), it was permissible to define hyperuricemia differently in different studies, but only studies with the mean serum urate level >7 mg/dl at baseline were included into our study. Two independent authors (XL and DHL) screened titles and abstracts in duplicate to ascertain potential eligibility. The exclusion criteria were studies 1) with follow-up time <3 months, 2) that included patients with end-stage renal disease, and 3) that included patients with kidney transplants. Potential eligible articles identified by either author subsequently underwent full-text review. Reasons for excluding articles were simultaneously recorded. Subsequently, three authors (XL, DHL, and YXQ) evaluated the full text of each article to determine whether it should be included. All selected articles were imported into EndNote and utilized for final analyses.

### Outcomes

Primary outcomes were changes in eGFR, uric acid, creatinine, proteinuria, and blood pressure, while AEs were assessed as secondary outcomes.

### Data Extraction and Quality Assessment

Data were collected in duplicate (XL and DHL), and primary authors were contacted when additional clarification was required. Concrete data points included study information (authors, year of publication, country, study type, sample size, interventions, and comparison arms) and features of study subjects (age, sex, inclusion and exclusion criteria, and clinical outcomes). Two authors (XL and DUL) reciprocally evaluated the extracted data, while another author (YXQ) resolved all disagreements when a consensus was not reached. Two authors (XL and DUL) independently evaluated the quality of each pair of comparison using the Cochrane risk-of-bias tool (Higgins et al., 2011; Supplementary Table 1).

### Data Synthesis and Analysis

Network meta-analysis of different interventions was performed using “gemtc 0.8-7” and its dependent packages in R software (version 3.6.3, The R Foundation, https://www.r-project.org). A multiple treatment comparison was conducted using a Bayesian network framework with a Monte Carlo Markov chain (MCMC) model (Jansen et al., 2008). We simultaneously conducted four MCMC models, and the number of simulations was set up to 5,000, with the number of iterations set up to 20,000. To evaluate the overall heterogeneity of the model, we used parameter I (Reginato et al., 2012) to calculate the deviation of the size of the heterogeneity. Mean differences (MDs) with 95% credibility intervals (CrIs) were calculated for continuous variables. For binary variables, odds ratios (ORs) with 95% CrIs were logarithmically converted into MDs with 95% CrIs.

For pairwise meta-analysis, MDs and ORs were calculated with 95% confidence intervals. *p* < 0.05 was considered statistically different. Heterogeneity was examined using the Q-test and I statistic (Reginato et al., 2012). A random-effects model was used when studies were heterogeneous (*p* < 0.1 or I^2^ > 50%). Otherwise, the fixed-effects model was applied. All pairwise meta-analyses were conducted in R with the “meta 4.15-1” package.

## Results

### Literature Search Outcomes and Study Features

**FIGURE 1:**
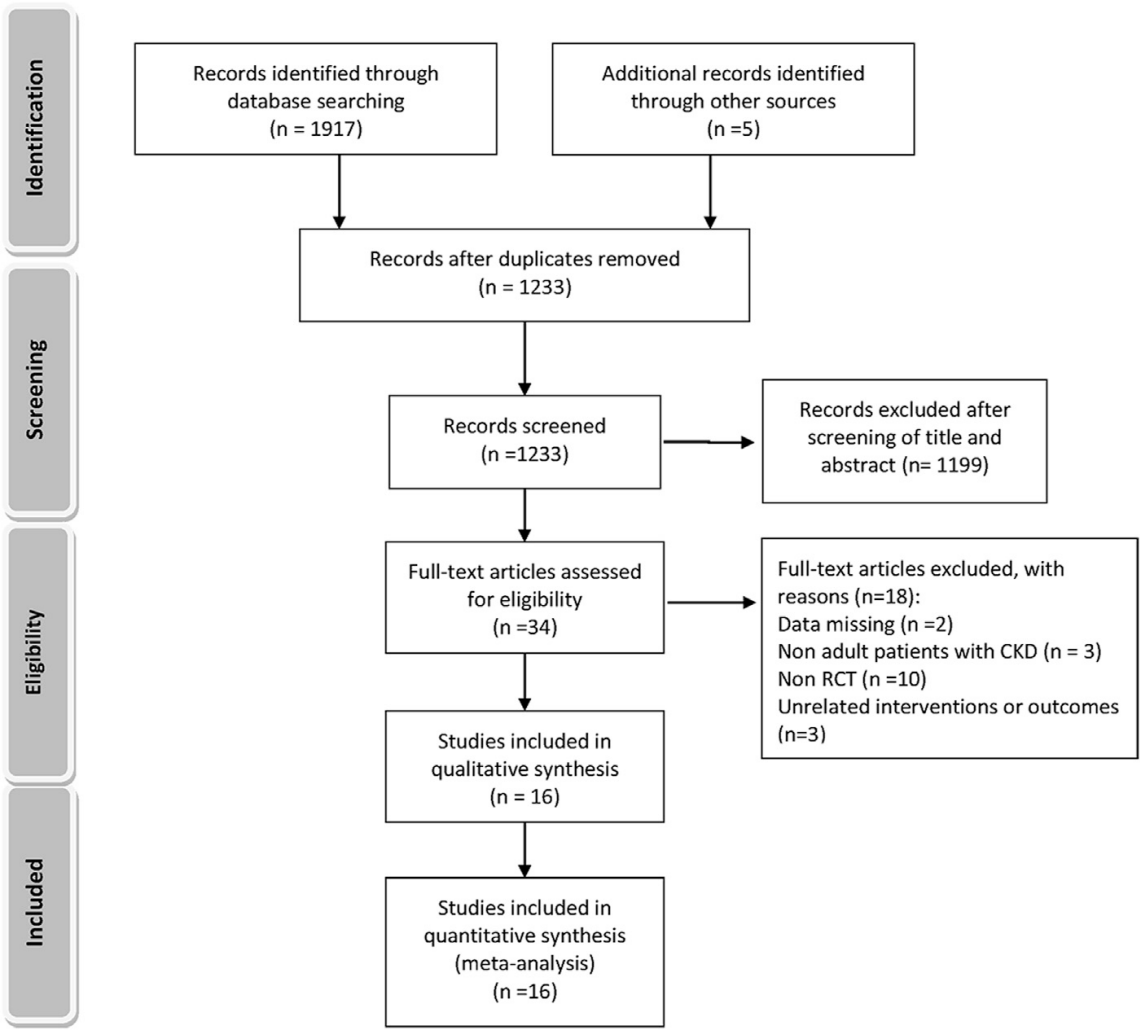
Flowchart of the study selection procedure.

**FIGURE 2:**
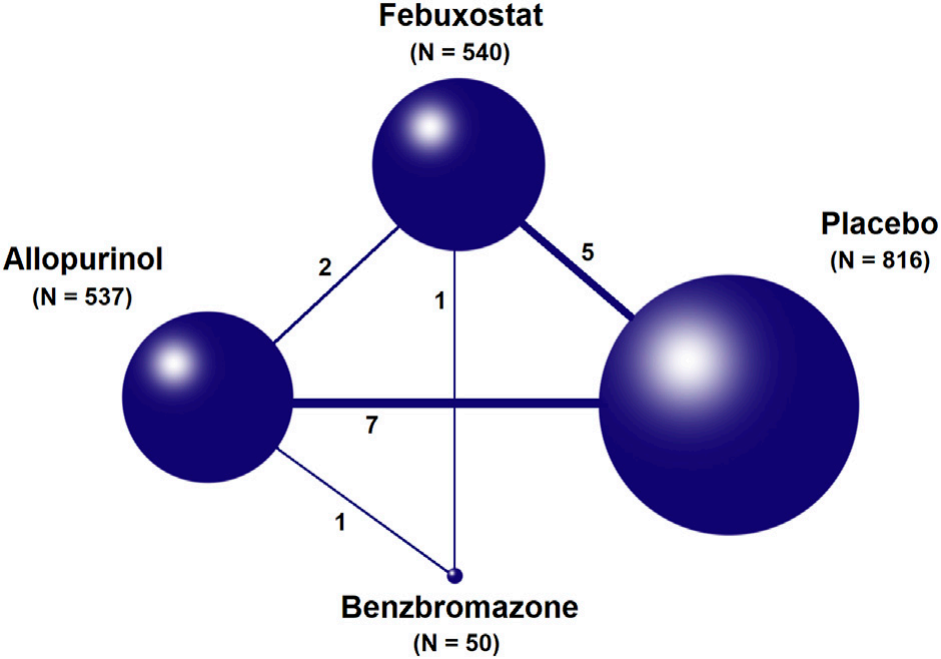
Network plots for relations between different therapies. The width of lines is proportional to the number of studies comparing every pair of interventions. The size of each circle is proportional to the sample size of each therapy.

A total of 1,233 titles were identified during the initial search, 1,202 of which were excluded upon screening of titles and abstracts. Following a full-text review of 31 studies, 16 RCTs involving 1,943 patients were included in the final network analysis (Figure 1) (Yu et al., 2018; Mukri et al., 2018; Kimura et al., 2018; Saag et al., 2016; Tanaka et al., 2015; Sircar et al., 2015; Sezai et al., 2015; Shi et al., 2012; Kao et al., 2011; Momeni et al., 2010; Goicoechea et al., 2010; Siu et al., 2006; Perez-Ruiz et al., 1999; Badve et al., 2020; Golmohammadi et al., 2017; Beddhu et al., 2016). Details on the 16 RCTs and the features of patients are summarized in Table 1 and Table 2, respectively. All studies included patients with CKD and hyperuricemia, except for one (Momeni et al., 2010) in which patients with normal uric acid levels were not excluded; therapeutic outcomes of febuxostat vs. benzbromarone, allopurinol, placebo, or usual therapy, and allopurinol vs. benzbromarone, or placebo/usual therapy were reported in one (Yu et al., 2018), two (Sezai et al., 2015; Tanaka et al., 2015), five (Sircar et al., 2015; Beddhu et al., 2016; Saag et al., 2016; Kimura et al., 2018; Mukri et al., 2018), one (Perez-Ruiz et al., 1999), and seven (Siu et al., 2006; Goicoechea et al., 2010; Momeni et al., 2010; Kao et al., 2011; Shi et al., 2012; Golmohammadi et al., 2017; Badve et al., 2020) studies, respectively. Relationships between different therapies are presented in network plots (Figure 2). The area of each circle represents the numbers of included patients, and the thickness of lines connecting them shows the number of articles comparing the two connected therapies.

**TABLE 1:** Study characteristics of included studies.

Author/year	Study type	Location	CKD criteria	Uric acid criteria	Intervention/control (n)	Doses of medication (mg/d)	Follow-up (months)
Badve et al. (2020)	RCT	Australia	eGFR 15–59	Mean SUA: 8.2	Allopurinol (182)	100–300	26
					Placebo (181)	-	
Yu et al. (2018)	RCT	China	eGFR 20–60	Gout or SUA ≥ 8.0 mg/dl	Febuxostat (33); benzbromarone (33)	20–80	12
						25–100	
Mukri et al. (2018)	RCT	Malaysia	eGFR 15–60	SUA ≥ 400 μmol/L	Febuxostat (47)	40	6
					No treatment (46)	-	
Kimura et al. (2018)	RCT	Japan	CKD3	SUA: 7.0–10.0 mg/dl	Febuxostat (219)	10	27
					Placebo (222)	-	
Golmohammadi et al. (2017)	RCT	Iran	eGFR 15–60	SUA > 6 mg/dl	Allopurinol (96)	100	12
					Placebo (100)	-	
Beddhu et al. (2016)	RCT	American	Diabetic nephrology	SUA ≥ 327 μmol/L (men)	Febuxostat (40)	80	6
				SUA ≥ 274 μmol/L (women)	Placebo (40)	-	
Saag et al. (2016)	RCT	American	eGFR 15–50	SUA > 7.0 mg/dl	Febuxostat (64)	30 twice daily or 40/80 mg once daily	12
					Placebo (32)	-	
Tanaka et al. (2015)	RCT	Japan	CKD3	SUA > 7.0 mg/dl	Febuxostat (21)	≤40	3
					Allopurinol (19)	50/100	
Sircar et al. (2015)	RCT	India	CKD3,4	SUA > 7 mg/dl	Febuxostat (45)	40	6
					Placebo (48)	-	
Sezai et al. (2015)	RCT	Japan	eGFR <60	Hyperuricemia	Febuxostat (71)	≤60	6
					Allopurinol (69)	≤300	
Shi et al. (2012)	RCT	China	IgA nephrology	SUA > 6 mg/dl (women)	Allopurinol (21)	100–300	6
				SUA > 7 mg/dl (men)	Usual therapy (19)	-	
Kao et al. (2011)	RCT	British	CKD3	Mean SUA: 7.23 mg/dl	Allopurinol (27)	100–300	9
					Placebo (26)	-	
Momeni et al. (2010)	RCT	Iran	Diabetic nephropathy	Mean SUA: 6.23 mg/dl	Allopurinol (20)	100	4
					Placebo (20)	-	
Goicoechea et al. (2010)	RCT	Spain	eGFR < 60 ml/min	Mean SUA: 7.6 mg/dl	Allopurinol (57) usual therapy (56)	100	24
						-	
Siu et al. (2006)	RCT	Hong Kong	Proteinuria > 500 g/d	SUA > 7.6 mg/dl	Allopurinol (25)	100–300	12
			And/or Cr > 120 mol/L		Usual therapy (26)	-	
Perez-Ruiz et al. (1999)	RCT	Spain	CCr < 80	Gouty arthritis	Allopurinol (19) benzbromarone (17)	100–300	9
						100–150	

RCT, randomized controlled trial; CKD, chronic kidney disease; eGFR (ml/min/1.73 m^2^), estimated glomerular filtration rate; Cr, creatinine; Ccr (ml/min/1.73 m^2^), clearance of creatinine.

**TABLE 2:** Baseline characteristics of included patients.

Author/year	Participants (N)	Intervention/control (N)	Gender (% male)	Mean age (SD)	Kidney function	Baseline SUA (mg/dl)
Badve et al. (2020)	363	Allopurinol (182)	62	62.3 ± 12.6	eGFR 31.6 ± 11.7	8.2 ± 1.8
		Placebo (181)	64	62.6 ± 12.9	31.9 ± 12.4	8.2 ± 1.7
Yu et al. (2018)	66	Febuxostat (33); benzbromarone (33)	75.8	59.5 ± 9	eGFR 38.5 ± 13.1	9.6 ± 1.86
			63.3	62.3 ± 7.6	41.2 (29.9–49.1)	8.87 ± 1.07
Mukri et al. (2018)	93	Febuxostat (47)	53.2	64 ± 10	eGFR 26.2 ± 14.3	9.07 ± 1.75
		Usual therapy (46)	54.3	67 ± 6	28.2 ± 19.8	9.03 ± 1.19
Kimura et al. (2018)	441	Febuxostat (219)	77.6	65.3 ± 11.8	eGFR 45.2 ± 9.5	7.8 ± 0.9
		Placebo (222)	77.0	65.4 ± 12.3	44.9 ± 9.7	7.8 ± 0.9
Golmohammadi et al. (2017)	296	Allopurinol (96)	55.2	NR	eGFR 44.53 ± 15.74	7.86 ± 1.36
		Placebo (100)	54		44.44 ± 16.03	7.75 ± 1.19
Beddhu et al. (2016)	80	Febuxostat (40)	60	67 ± 10	eGFR 52.2 ± 15.3	7.16 ± 1.50
		Placebo (40)	70	68 ± 11	54.8 ± 19.0	7.09 ± 1.19
Saag et al. (2016)	96	Febuxostat (64)	79.7	65.51 ± 9.84	eGFR 34.1	10.36 ± 1.56
		Placebo (32)	81.3	66.3 ± 12.05	29.31	10.8 ± 1.96
Tanaka et al. (2015)	40	Febuxostat (21)	90.5	70.1 ± 9.5	eGFR 41.8 ± 12	7.75 ± 0.84
		Allopurinol (19)	84.2	66.1 ± 7	47.4 ± 11	8.18 ± 1.11
Sircar et al. (2015)	93	Febuxostat (45)	64.4	56.22 ± 10.87	eGFR 31.5 ± 13.6	9 ± 2
		Placebo (48)	77.1	58.42 ± 14.52	32.6 ± 11.6	8.2 ± 1.1
Sezai et al. (2015)	140	Febuxostat (71)	81.7	67.4 ± 9.7	eGFR 40.11 ± 10.4	8.61 ± 0.96
		Allopurinol (69)	82.6	66.4 ± 10.8	41.5 ± 10.6	8.56 ± 0.98
Shi et al. (2012)	40	Allopurinol (21)	61.9	39.7 ± 10	eGFR 69.5 ± 26.5	7.9 ± 1.1
		Usual therapy (19)	47.4	40.1 ± 10.8	63.6 ± 27.5	7.8 ± 1.1
Kao et al. (2011)	53	Allopurinol (27)	59.3	70.6 ± 6.9	eGFR 44 ± 11	7.39 ± 1.51
		Placebo (26)	46.2	73.7 ± 5.3	46 ± 9	7.06 ± 1.34
Momeni et al. (2010)	40	Allopurinol (20)	45	56.3 ± 10.6	Scr 1.3 ± 0.45	5.96 ± 1.21
		Placebo (20)	45	59.1 ± 10.6	1.5 ± 0.6	6.5 ± 2.2
Goicoechea et al. (2010)	113	Allopurinol (57) usual therapy (56)	NR	72.1 ± 7.9	40.8 ± 11.2	7.8 ± 2.1
			NR	71.4 ± 9.5	39.5 ± 12.4	7.3 ± 1.6
Siu et al. (2006)	51	Allopurinol (25)	16	47.7 ± 12.9	Proteinuria 2.39 ± 2.88	9.75 ± 1.18
		Usual therapy (26)	57.7	48.8 ± 16.8	2.39 ± 2.2	9.92 ± 1.68
Perez-Ruiz et al. (1999)	36	Allopurinol (19) benzbromarone (17)	86.1 (overall)	67.3 ± 9.59	CCr 53.28 ± 16.67	8.96 ± 1.84
				60.9 ± 12.8	54.52 ± 17.47	9.35 ± 1.96

RCT, randomized controlled trial; NR, not reported; eGFR (ml/min/1.73 m^2^), estimated glomerular filtration rate; Scr (mg/dl), serum creatinine; Ccr(ml/min/1.73 m^2^), clearance of creatinine; proteinuria, g/d.

### Outcomes

#### Uric Acid

**FIGURE 3:**
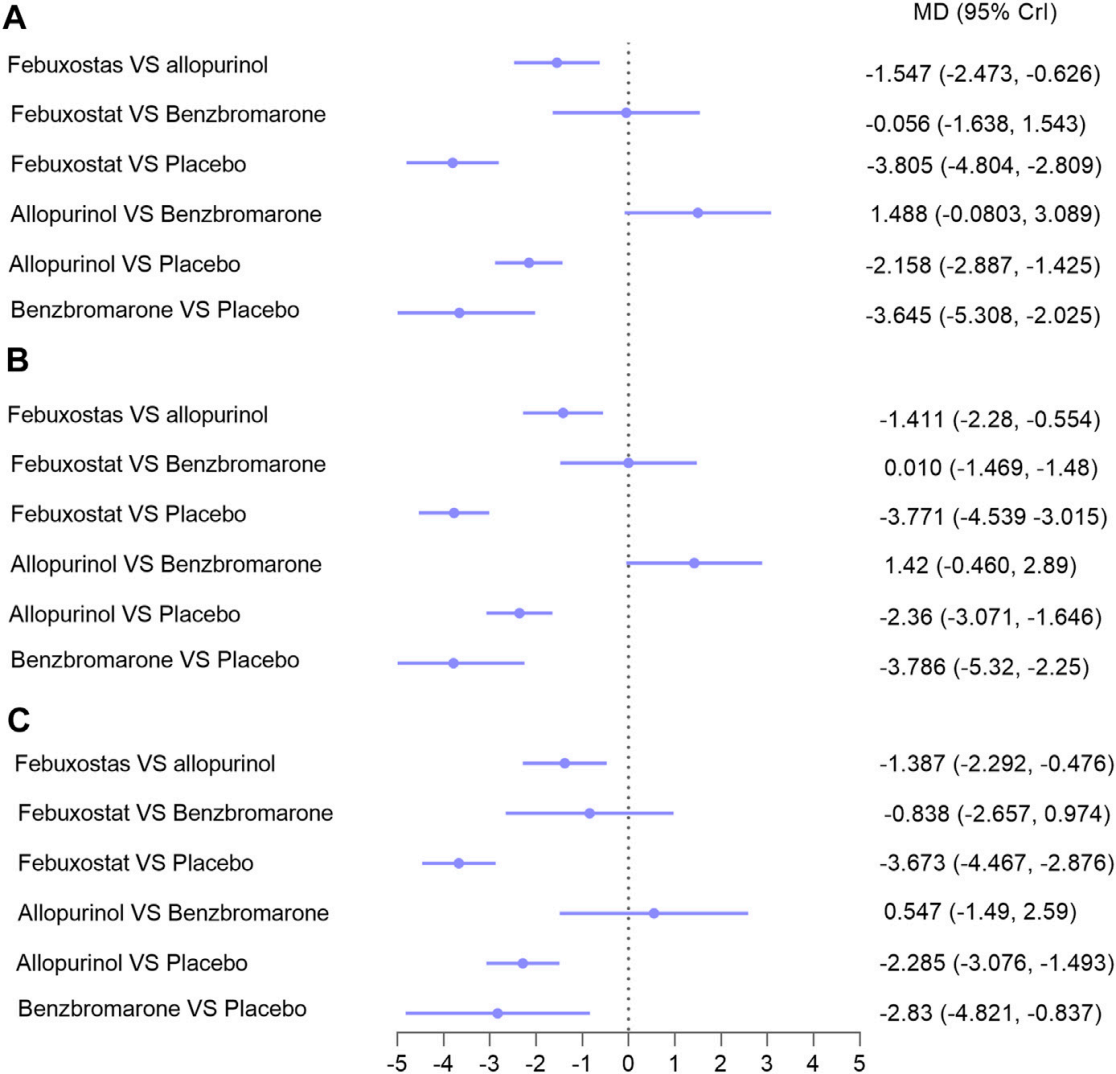
Network meta-analysis results for lowering uric acid in **(A)** patients with CKD; **(B)** patients with hyperuricemia and CKD; **(C)** patients with hyperuricemia and eGFR < 60 ml/min/1.73 m^2^. Abbreviations: MD, mean difference; CrIs, credible intervals; CKD, chronic kidney disease; eGFR (ml/min/1.73 m^2^), estimated glomerular filtration rate.

Sixteen studies with 1,672 patients (271 patients were lost to follow-up or death) provided data on ULT. Network meta-analysis demonstrated that febuxostat, allopurinol, and benzbromarone were statistically superior to placebo at lowering urate, while febuxostat was associated with superior improvement in uric acid levels compared with allopurinol (MD: −1.547; 95% CrI: −2.473 to −0.626) (Figure 3A, I = 3%) (Reginato et al., 2012). No significant differences were found when benzbromarone was compared with febuxostat and allopurinol (Figure 3A). When we only included studies with patients with CKD and hyperuricemia (Yu et al., 2018; Mukri et al., 2018; Kimura et al., 2018; Saag et al., 2016; Tanaka et al., 2015; Sircar et al., 2015; Sezai et al., 2015; Shi et al., 2012; Kao et al., 2011; Goicoechea et al., 2010; Siu et al., 2006; Perez-Ruiz et al., 1999; Badve et al., 2020; Golmohammadi et al., 2017; Beddhu et al., 2016) (Reginato et al., 2012) (Figure 3B, I = 4%), or patients with eGFR < 60 ml/min/1.73 m^2^ and hyperuricemia (Yu et al., 2018; Mukri et al., 2018; Kimura et al., 2018; Saag et al., 2016; Tanaka et al., 2015; Sircar et al., 2015; Sezai et al., 2015; Kao et al., 2011; Goicoechea et al., 2010; Badve et al., 2020; Golmohammadi et al., 2017) (Reginato et al., 2012) (Figure 3C, I = 5%), results were consistent.

Subgroup analysis was performed based on follow-up time (6 months). Data on patients with over 6 months of follow-up were available in nine studies with 1,150 patients. The difference between febuxostat and allopurinol was not statistically significant in these patients (Supplementary Figure 1b, indirect comparison); this result had low certainty of evidence and lacked credibility because of a lack of head-to-head comparison studies. The remaining results were consistent with those described above (Supplementary Figures 1a,b; I ≤10%) (Reginato et al., 2012).

#### Progression of Chronic Kidney Disease

**FIGURE 4:**
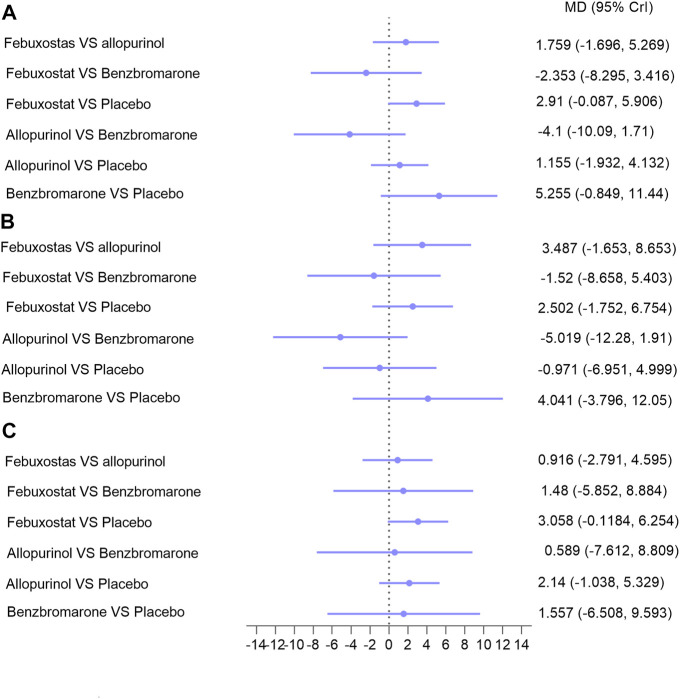
Network meta-analysis results for lowering eGFR decline in **(A)** patients with CKD; **(B)** patients with hyperuricemia and CKD; **(C)** patients with hyperuricemia and eGFR < 60 ml/min/1.73 m^2^. Abbreviations: MD, mean difference; CrIs, credible intervals; CKD, chronic kidney disease; eGFR (ml/min/1.73 m2), estimated glomerular filtration rate.

Indicators of CKD progression, including the change in eGFR, proteinuria, and serum creatinine, were analyzed. Regarding eGFR, 14 trials with 1,594 patients were available for assessment (Yu et al., 2018; Mukri et al., 2018; Kimura et al., 2018; Saag et al., 2016; Tanaka et al., 2015; Sircar et al., 2015; Sezai et al., 2015; Shi et al., 2012; Kao et al., 2011; Goicoechea et al., 2010; Perez-Ruiz et al., 1999; Badve et al., 2020; Golmohammadi et al., 2017; Beddhu et al., 2016). Febuxostat, allopurinol, and benzbromarone did not exert superior effects on improving eGFR over placebo in overall patients (Figure 4, I = 5%) (Reginato et al., 2012), or in patients with follow-up time over or less than 6 months (Supplementary Figure 2). Similarly, no differences were found between the three interventions regarding improvement of eGFR (Figure 4). Similar results were found with serum creatinine (seven studies with 620 patients; Supplementary Figures 3a–c) and proteinuria (five studies with 290 patients; Supplementary Figure 3d). However, febuxostat tended to be superior to allopurinol on lowering the decline of both eGFR (Figure 4) and proteinuria (Supplementary Figure 3d), and the difference did not reach statistical significance.

#### Blood Pressure

**FIGURE 5:**
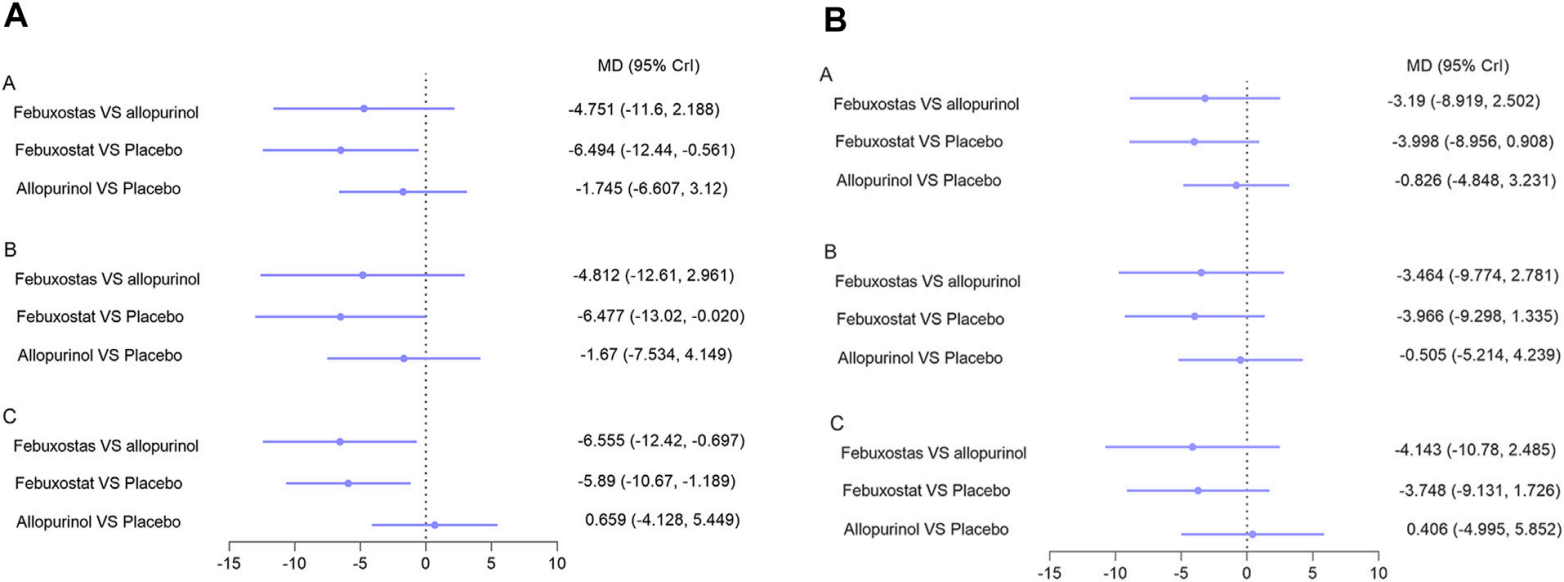
Network meta-analysis results for lowering **(A)** systolic blood pressure and **(B)** diastolic blood pressure. Note: **A**: patients with CKD; **B**: patients with hyperuricemia and CKD; **C**: patients with hyperuricemia and eGFR < 60 ml/min/1.73 m^2^. Abbreviations: MD, mean difference; CrI, credible intervals; CKD, chronic kidney disease; eGFR (ml/min/1.73 m^2^), estimated glomerular filtration rate.

Blood pressure analysis included data from nine studies with 1,045 patients (Kimura et al., 2018; Tanaka et al., 2015; Sircar et al., 2015; Kao et al., 2011; Momeni et al., 2010; Goicoechea et al., 2010; Siu et al., 2006; Badve et al., 2020; Beddhu et al., 2016). Overall, network meta-analysis indicated that febuxostat was superior to placebo with respect to control systolic blood pressure, while allopurinol and benzbromarone were not found to have superior effects than placebo (Figure 5A, I^2^ = 7%, Reginato et al., 2012, respectively). Notably, febuxostat was associated with better control of systolic blood pressure than allopurinol in patients with eGFR < 60 ml/min/1.73 m^2^ and hyperuricemia (MD: −6.555, 95% CrI: −12.42 to −0.69) (Figure 5A). No differences were found among these interventions in terms of controlling diastolic blood pressure (Figure 5B).

#### Adverse Events

AEs including deterioration of kidney function (eight studies with 1,200 patients), liver dysfunction (seven studies with 939 patients), cardiovascular events (six studies with 1,141 patients), gastrointestinal symptoms (eight studies with 1,270 patients), and hypersensitivity (eight studies with 1,117 patients) were analyzed. There are no differences found in these AEs among the four interventions (Supplementary Figures 4, 5); febuxostat showed a tendency to be safer with respect to hypersensitivity, renal impairment, and liver dysfuction than allopurinol, but the difference was not statistically significant (Supplementary Figure 4).

### Sensitivity Analysis, Subgroup Analysis, and Risk of Bias

Sensitivity analysis was performed by excluding studies that included patients without hyperuricemia and studies with eGFR ≥ 60 ml/min/1.73 m^2^. Furthermore, a subgroup analysis was performed based on follow-up time (≤6 or >6 months). As described above, sensitivity and the subgroup analysis did not affect our findings to an appreciable degree, highlighting the robustness of our study. The risk of bias for each study is shown in Supplementary Table 1. Overall, the risk of participant or investigator blinding was relatively high, while the risks of other parameters were low or unclear.

## Discussion

It is well established that reduced kidney function is accompanied by increased serum urate levels resulting from decreased kidney clearance (Reginato et al., 2012). However, it is increasingly suspected that rising urate levels play a role in the pathogenesis and progression of CKD, and are not simply a marker of kidney disease (Uchida et al., 2015). Whether urate-lowering agents have the capacity to slow the progression of CKD has not been fully elucidated, and studies examining differences in urate-lowering agents such as febuxostat, allopurinol, and benzbromarone are very limited.

This is the first systematic review and network meta-analysis to investigate differences in effectiveness of three commonly used drugs at promoting renal protection and urate reduction in patients with CKD and hyperuricemia. We found that febuxostat can lower serum uric acid and control blood pressure better than allopurinol and benzbromarone. However, the three agents were found to not exert renoprotective effects, including improvement in eGFR, creatinine, or proteinuria. Similar observations were made in subgroup analyses stratified by the CKD stage and follow-up time.

Our findings are consistent with select RCTs in which ULT was shown to have no obvious effects on renal function (Perez-Ruiz et al., 1999; Shi et al., 2012; Beddhu et al., 2016; Saag et al., 2016; Kimura et al., 2018; Yu et al., 2018; Badve et al., 2020). A recent randomized, double-blinded, placebo-controlled study across 31 centers published in the New England Journal of Medicine also concluded that ULT with allopurinol did not improve the decline in eGFR compared with placebo in patients with CKD (Badve et al., 2020). Interestingly, in a prospective cohort study, allopurinol and febuxostat also failed to exert renoprotective effects, even though hyperuricemia was found to be an independent risk factor for CKD advancement (Oh et al., 2019). Conversely, ULT was shown to improve renal function in meta-analyses by Zeng et al. (2018) and Kanji et al. (2015). However, Zeng et al. included a retrospective analysis, and an evaluation in the febuxostat group that lacked data on eGFR was included in error. The study by Kanji et al. analyzed trials with short follow-up time (less than 2 months), and they incorporated Chinese databases, resulting in several non-English RCTs. Notably, febuxostat was also shown to be effective at reducing the risk of CKD progression in CKD populations with hyperuricemia in certain prospective or retrospective cohort studies (Chou et al., 2018; Liu et al., 2019; Yang, 2020); in our analysis, febuxostat showed a tendency to be superior to allopurinol on lowering the decline of eGFR and increment of proteinturia, although the difference is not statistically significant. These conflicting results highlight the need for large, double-blind RCTs to study the differences in renoprotective effects of urate-lowering agents.

We also found that febuxostat was superior at lowering uric acid and controlling blood pressure. Inadequate dosing of allopurinol may explain its poor effectiveness, as the dose of allopurinol in most included studies was less than 300 mg/d (Table 1) because of concerns over possible fetal side effects. Febuxostat, a more recent xanthine oxidase inhibitor (approved by the Food and Drug Administration in 2009), may be a reasonable choice for hyperuricemic patients with CKD as it is cost-effective and well tolerated (Gandhi et al., 2015; Tiku et al., 2018). Furthermore, febuxostat was proven to be more potent for lowering urate, and dose adjustment is not required in CKD patients (Shibagaki et al., 2014; Hira et al., 2015). Therefore, febuxostat may be more effective for lowering urate. Benzbromarone is not recommended for patients with eGFR < 30 ml/min (Richette et al., 2016) and was withdrawn from the United States and several European countries (Wang et al., 2017). Only two studies with 102 patients provided data on benzbromarone. Therefore, the evidence had low certainty and lacked credibility.

Compared with previous meta-analyses (Kim et al., 2017; Liu et al., 2018; Lin et al., 2019; Hu and Brown, 2020), the present report had several strengths. The available evidence was searched comprehensively, only RCTs with follow-up time over 3 months were included, and heterogeneity was very low (≤10%). Additionally, inclusion criteria were restricted to patients with CKD and hyperuricemia. Allopurinol, febuxostat, and benzbromarone were compared directly rather than with placebo only or with placebo/other agents collectively. Futhermore, we performed subgroup analyses according to the CKD stage and follow-up time. Indicators of renal function, including eGFR, creatinine, and proteinuria, were comprehensively evaluated, and AEs (including deterioration of renal or liver function, cardiovascular events, gastrointestinal symptoms, and hypersensitivity) were evaluated. Overall, differences in the effects of febuxostat, allopurinol, and benzbromarone on lowering urate, renal protection, blood pressure control, and AEs in hyperuricemic patients with CKD were compared for the first time, and the number of included RCTs and patients was relatively substantial.

This study also had limitations. First, the methodological quality of trials was suboptimal, as allocation concealment was unclear in most studies because of unexhaustive description of methods, and double-blinding was rated as a high risk in one-third of the trials analyzed. Second, evaluation of renal function focused on eGFR (14 trials with 1,594 patients), creatinine (seven studies with 620 patients), and proteinuria (five studies with 290 patients), while evaluation of progression to end-stage renal disease was insufficient (no data). Finally, while our findings have a reference value, they should not be applied to patients with only CKD or hyperuricemia, as the patients included in our study suffered from both hyperuricemia and CKD.

## Conclusion

There is insufficient evidence to support the renoprotective effects of the three urate-lowering agents in CKD patients with hyperuricemia. Regarding its urate-lowering effect, febuxostat appears to be a satisfactory alternative to allopurinol and benzbromarone. It is more effective at lowering serum uric acid and controlling blood pressure in hyperuricemic patients with CKD. Interestingly, febuxostat shows a tendency to be superior to allopurinol on lowering the decline of eGFR and increment of proteinturia, although the difference does not reach a statistical significance; large, double-blind RCTs that study differences in the renoprotective effects of different urate-lowering agents are necessary.

## Data Availability

The original contributions presented in the study are included in the article/Supplementary Material; further inquiries can be directed to the corresponding author.
