# Supplementary material for: Effectiveness of Drug Treatments for Lowering Uric Acid on Renal Function in Patients With Chronic Kidney Disease and Hyperuricemia: A Network Meta-Analysis of Randomized Controlled Trials
Source: Front Pharmacol. 2021 Aug 3;12:690557. doi: 10.3389/fphar.2021.690557 (PMC8369347; doi:10.3389/fphar.2021.690557)
Supplement: Supplementary file 1 [file DataSheet2.DOCX]

Supplemental Table 1. The risk of bias of randomized controlled trials.

| Study ID | Random sequence generation | Allocation concealment | Participant blinding | Investigator binding | Incomplete outcome data | Selective reporting | Other source of bias |
| --- | --- | --- | --- | --- | --- | --- | --- |
| Sunil V 2020 | low | low | unclear | unclear | low | low | low |
| Yu 2018 | low | unclear | unclear | unclear | low | low | low |
| Kimura 2018 | low | low | low | low | low | low | unclear |
| Mukri 2018 | low | unclear | high | high | low | low | low |
| Golmohammadi 2017 | unclear | unclear | unclear | unclear | low | low | high |
| Beddhu 2016 | low | low | low | low | low | low | low |
| Saag 2016c | high | low | low | low | low | low | unclear |
| Sircar 2015 | low | low | low | low | high | low | low |
| Tanaka 2015 | unclear | low | high | high | low | low | low |
| Sezai 2014 | low | unclear | low | high | low | low | low |
| Shi 2012 | low | low | high | low | low | low | low |
| Kao 2011 | unclear | unclear | low | low | low | low | low |
| Momeni 2010 | unclear | unclear | low | low | low | low | low |
| Giocoechea 2010 | low | unclear | high | low | low | low | unclear |
| Siu 2006 | low | unclear | high | high | low | low | unclear |
| Perez-Ruiz 1999 | unclear | unclear | high | high | low | low | low |
