# Supplementary material for: Effectiveness of Drug Treatments for Lowering Uric Acid on Renal Function in Patients With Chronic Kidney Disease and Hyperuricemia: A Network Meta-Analysis of Randomized Controlled Trials
Source: Front Pharmacol. 2021 Aug 3;12:690557. doi: 10.3389/fphar.2021.690557 (PMC8369347; doi:10.3389/fphar.2021.690557)

**Supplemental figure 1.** Network meta-analysis result (MD, 95%CrI) for lowering uric acid in those with (a) less than or equal to 6 months of follow-up time; and (b) more than 6 months of follow-up time.

**Note:** A: patients with CKD; B: patients with hyperuricemia and CKD; C: patients with hyperuricemia and eGFR < 60ml/min/1.73m2.

**Abbreviations:** MD: mean difference; CrI: credible intervals; CKD: chronic kidney disease. eGFR (ml/min/1.73m2): estimated glomerular filtration rate;


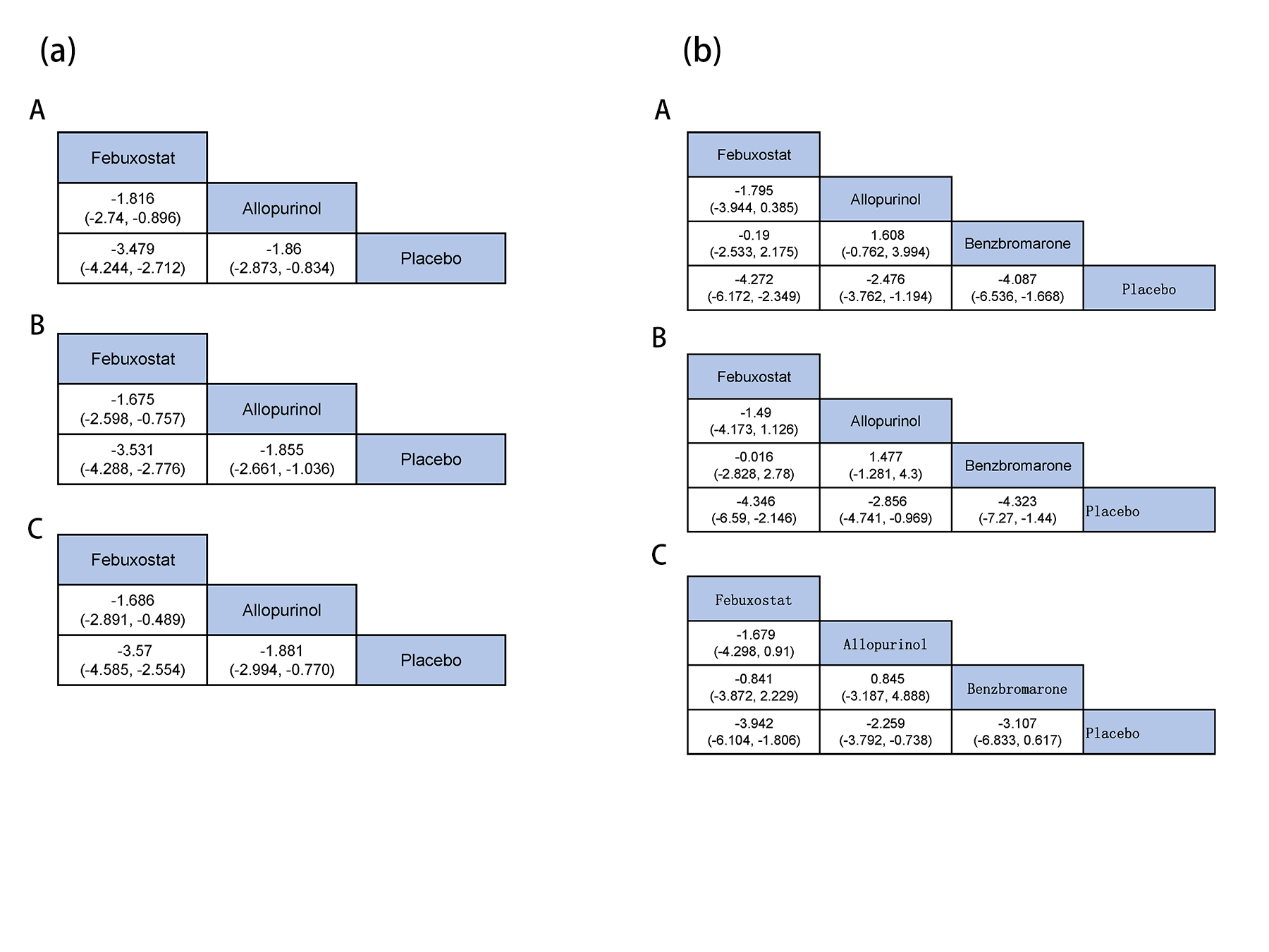


**Supplemental figure 2.** Network meta-analysis result (MD, 95%CrI) for lowering eGFR decline in those with (a) less than or equal to 6 months of follow-up time; and (b) more than 6 months of follow-up time.

**Note**: A: patients with CKD; B: patients with hyperuricemia and CKD; C: patients with hyperuricemia and eGFR < 60ml/min/1.73m2.

**Abbreviations:** MD: mean difference; CrI: credible intervals; CKD: chronic kidney disease. eGFR (ml/min/1.73m2): estimated glomerular filtration rate;


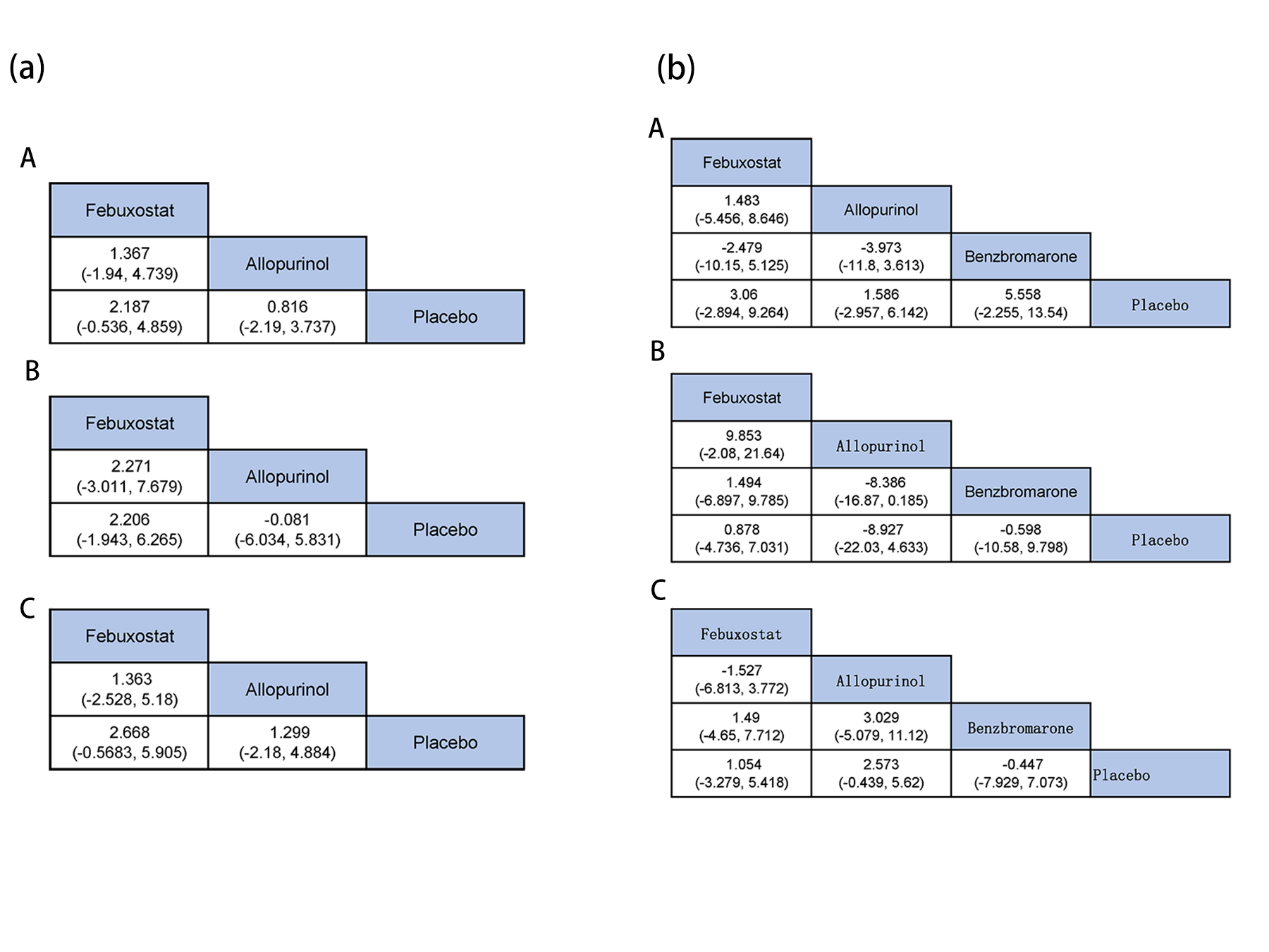


**Supplemental figure 3.**  Network meta-analysis result (MD, 95%CrI) for change of creatinine in (a) overall patients; (b) patients with less than or equal to 6 months follow-up time; (c) patients with more than 6 months follow-up time and (d) change of proteinuria

**Note**: A: patients with CKD; B: patients with hyperuricemia and eGFR < 60ml/min/1.73m2.

**Abbreviations:** MD: mean difference; CrI: credible intervals; CKD: chronic kidney disease. eGFR (ml/min/1.73m2): estimated glomerular filtration rate;


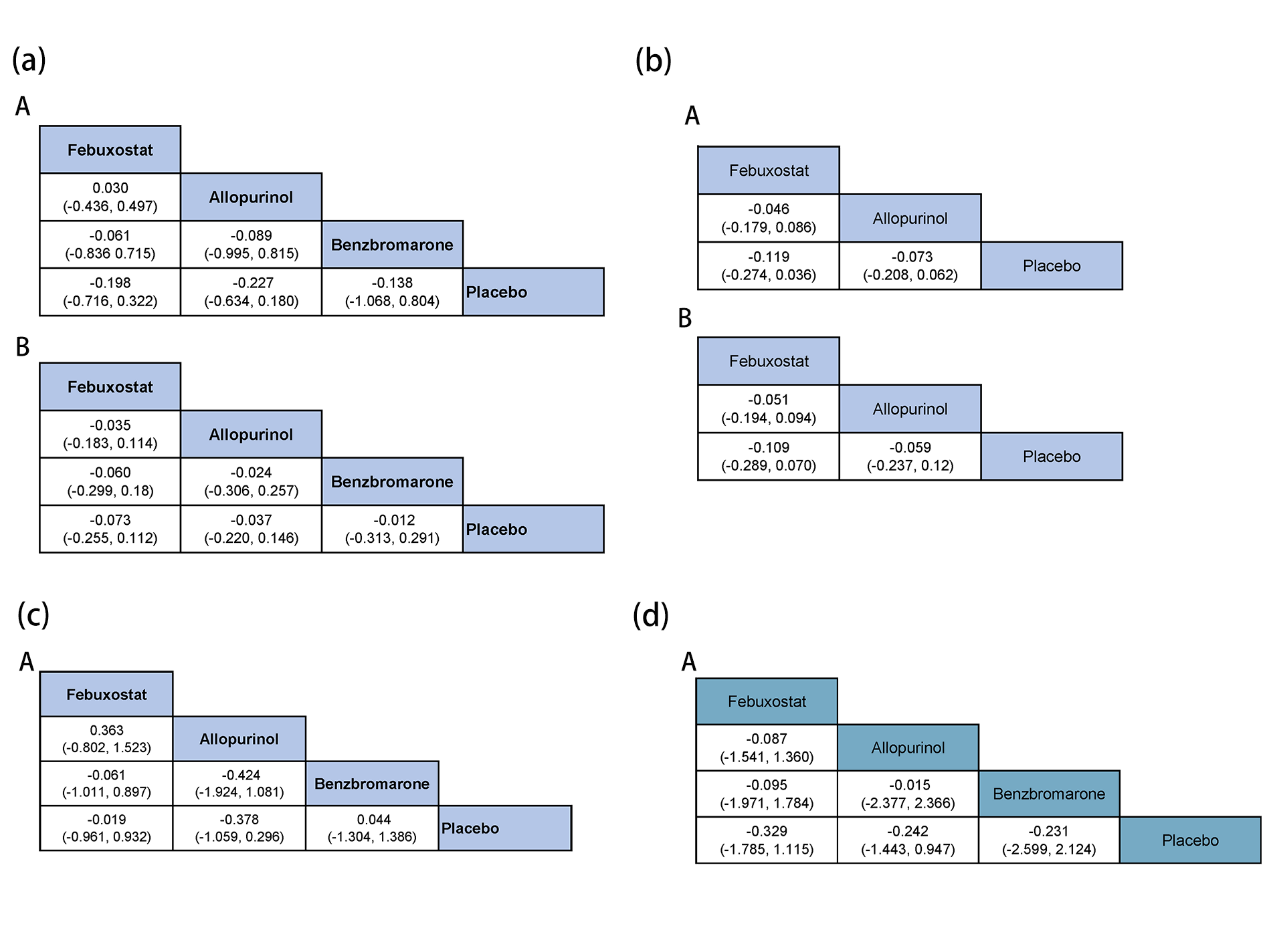


**Supplemental figure 4.**  Network meta-analysis result (MD, 95%CrI) for adverse events, including (a) hypersensitivity (skin rash or eruption etc.) (b) renal impairment; (c) liver dysfunction and (d) cardiovascular events.

**Abbreviations:** MD: mean difference; CrI: credible intervals; CKD: chronic kidney disease.


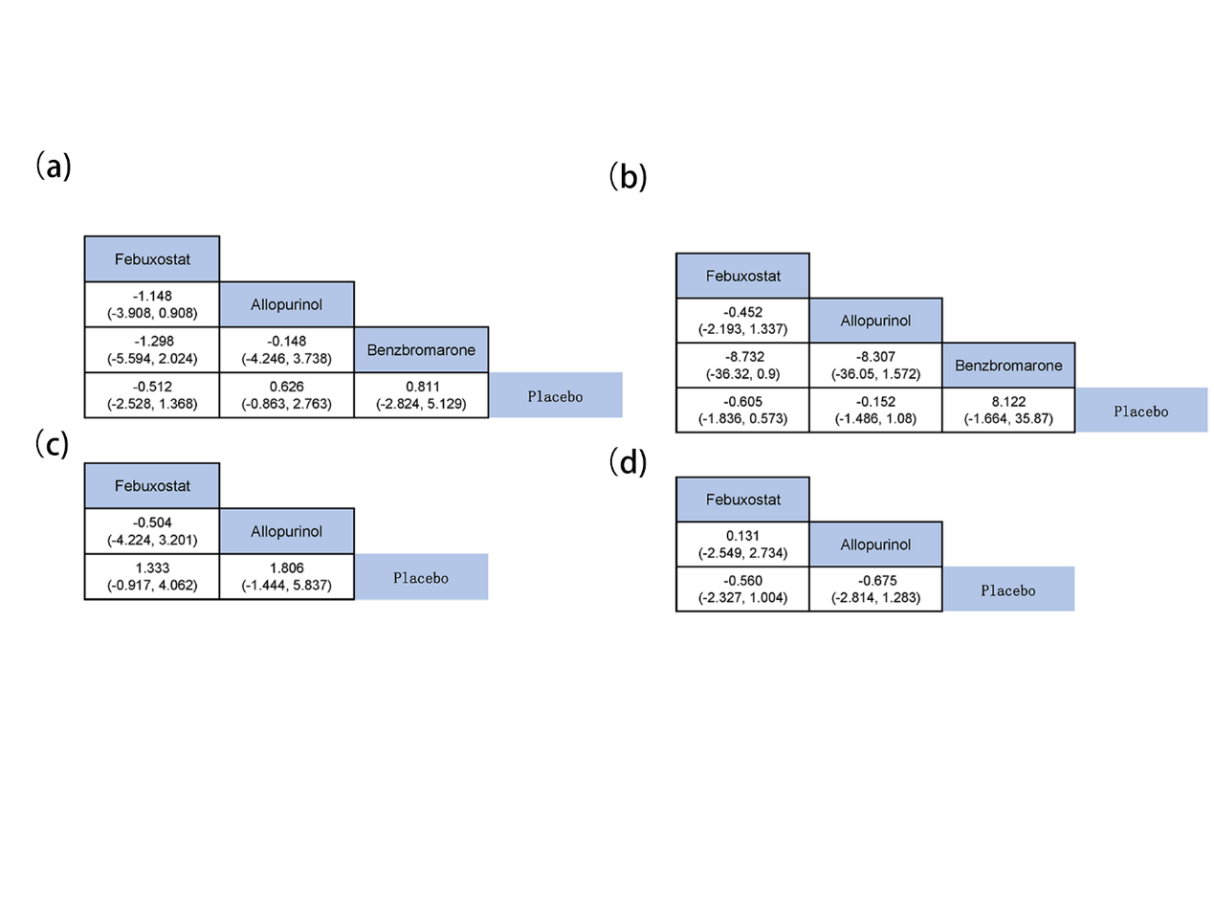


**Supplemental figure 5.**  Network meta-analysis result (MD, 95%CrI) for gastrointestinal symptoms in patients with CKD and hyperuricemia.

**Abbreviations:** MD: mean difference; CrI: credible intervals; CKD: chronic kidney disease.


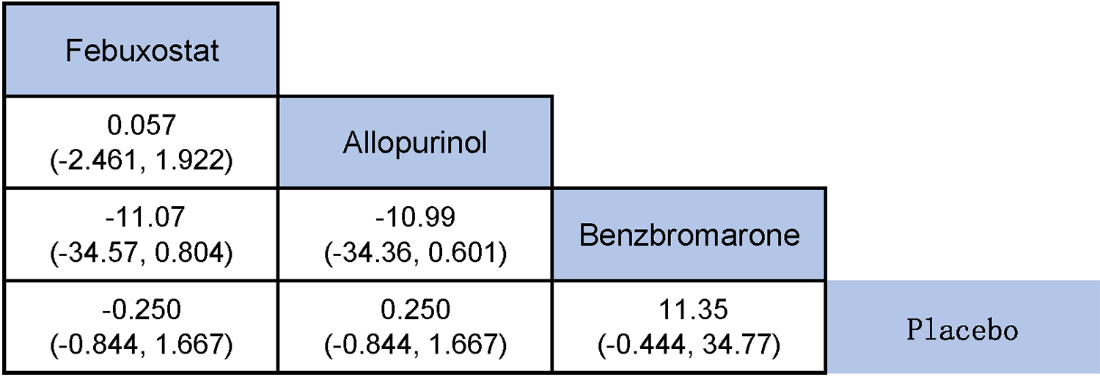

Supplement: Supplementary file 2 [file DataSheet1.DOCX]
